# α-Mangostin Alleviated HIF-1α-Mediated Angiogenesis in Rats With Adjuvant-Induced Arthritis by Suppressing Aerobic Glycolysis

**DOI:** 10.3389/fphar.2021.785586

**Published:** 2021-12-20

**Authors:** Tian-Tian Jiang, Chao-Fan Ji, Xiu-Ping Cheng, Shao-Fei Gu, Rui Wang, Yan Li, Jian Zuo, Jun Han

**Affiliations:** ^1^ Department of Traditional Chinese Medicine, The First Affiliated Hospital of Wannan Medical College (Yijishan Hospital), Wuhu, China; ^2^ Anhui Provincial Engineering Laboratory for Screening and Re-Evaluation of Active Compounds of Herbal Medicines in Southern Anhui, Wuhu, China; ^3^ Xin’an Medicine Research Center, Wannan Medical College, Wuhu, China; ^4^ Research Center of Integration of Traditional Chinese and Western Medicine, Wannan Medical College, Wuhu, China; ^5^ Key Laboratory of Non-Coding RNA Transformation Research of Anhui Higher Education Institution, Wannan Medical College, Wuhu, China

**Keywords:** glycometabolism, rheumatoid arthritis (RA), hypoxia inducible factor-1α (HIF-1α), metabolism reprogramming, oxidative stress MAN suppressed glycolysis-related angiogenesis

## Abstract

A previously validated anti-rheumatic compound α-mangostin (MAN) shows significant metabolism regulatory effects. The current study aimed to clarify whether this property contributed to its inhibition on synovial angiogenesis. Male wistar rats with adjuvant-induced arthritis (AIA) were orally treated by MAN for 32 days. Afterwards, biochemical parameters and cytokines in plasma were determined by corresponding kits, and glycometabolism-related metabolites were further accurately quantified by LC-MS method. Anti-angiogenic effects of MAN were preliminarily assessed by joints based-immunohistochemical examination and matrigel plug assay. Obtained results were then validated by experiments *in vitro*. AIA-caused increase in circulating transforming growth factor beta, interleukin 6, hypoxia inducible factor-1 alpha (HIF-1α) and vascular endothelial growth factor (VEGF) in blood and local HIF-1α/VEGF expression in joints was abrogated by MAN treatment, and pannus formation within matrigel plugs implanted in AIA rats was inhibited too. Scratch and transwell assays revealed the inhibitory effects of MAN on human umbilical vein endothelial cells (HUVECs) migration. Furthermore, MAN inhibited tubule formation capability of HUVECs and growth potential of rat arterial ring-derived endothelial cells *in vitro*. Meanwhile, MAN eased oxidative stress, and altered glucose metabolism *in vivo*. Glycolysis-related metabolites including glucose 6-phosphate, fructose 6-phosphate, 3-phosphoglyceric acid and phosphoenolpyruvic acid in AIA rats were decreased by MAN, while the impaired pyruvate-synthesizing capability of lactate dehydrogenase (LDH) was recovered. Consistently, MAN restored lipopolysaccharide-elicited changes on levels of glucose and LDH in HUVECs culture system, and exerted similar effects with LDH inhibitor stiripentol on glycometabolism and VEGF production as well as tubule formation capability of HUVECs. These evidences show that MAN treatment inhibited aerobic glycolysis in AIA rats, which consequently eased inflammation-related hypoxia, and hampered pathological neovascularization.

## Introduction

Rheumatoid arthritis (RA) is the most common autoimmune disease. Its incidence is as high as 0.5–1% worldwide. Without effective interventions, it will eventually lead to disabilities (Kim et al., 2017). It has been well known that self-tolerance breakdown directly accounts for RA-related abnormal immune responses, and provokes most pathological changes (Kim et al., 2017). Hence, disease-modifying anti-rheumatic drugs (DMARDs) with immunoregulatory properties have been adopted as the mainstay of RA treatments for decades. But our knowledge about RA immunity is still insufficient, which limits the potential of DMARDs-based regimens (Smolen and Aletaha, 2008). Due to the same reason, we cannot expect a great leap ahead about the development and application of biological anti-rheumatic reagents in near future (Zago et al., 2020). At the present stage, efficiently controlling arthritic manifestations remains a priority of RA therapies. Because RA is typically characterized by chronic inflammation, glucocorticoids and nonsteroidal anti-inflammatory drugs are extensively used. However, effects of these anti-inflammatory reagents on joint degradation are weak, and they cannot substantially prevent arthritic progress in the long term (Zhou et al., 2019).

There are many players implicated in joints injuries, such as fibroblasts, immune cells, osteoclasts and so on. They have distinctly different physiological functions (Shiozawa et al., 2011). Thoroughly elucidating the mystery how joints degradation initiates and progresses in RA patients is very challenging. Nonetheless, one fact is clear. All these cells require stable nutrient supply, and the increased energy consumption always fuels pathological changes. Hence, the abnormal synovial neovascularization in joints directly or indirectly promotes arthritis progress by providing extra energy supply. Consistent to this notion, pannus is extensively observed in the joints of arthritic subjects, which has been identified as a hallmark of RA-related synovitis (Szekanecz et al., 2009; Leblond et al., 2017). By abrogating neovascularization, synovial hyperplasia and immune cells infiltration will be substantially eased. Numerous successful experiments confirm that this is a realistic and feasible strategy to treat joint damages (Leblond et al., 2017). Actually, many DMARDs possess this pharmacological property (He et al., 2010; Chu and Zhang, 2018). Hypoxia inducible factor-1 alpha/vascular endothelial growth factor (HIF-1α/VEGF) axis is the most investigated angiogenesis signaling (Ahluwalia and Tarnawski, 2012). Besides the activation of inflammatory pathways, metabolic alteration is another vital factor inducing HIF-1α expression (Eltzschig and Carmeliet, 2011; Ahluwalia and Tarnawski, 2012; Liu-Bryan, 2015). Theoretically, targeting either inflammation or metabolism will affect HIF-1α/VEGF signaling. Until now, most researches focus on the anti-inflammatory approaches, while clinical potentials of metabolic intervention approaches are still to be explored. In fact, there are many reagents with significant metabolism regulatory effects available nowadays. Among them, natural products are especially eye-catching because of the good safety profiles and long history of use.

Mangosteen is a tropical fruit native to south Asia. Its pericarp is traditionally used as a medicine to cure inflammation and pains in Thailand, India, Burma and southern China (Pedraza-Chaverri et al., 2008). As the main mangosteen-derived bioactive compound, α-mangostin (MAN) exhibits notable therapeutic potentials against tumor, inflammation, metabolic disorders and many other diseases (Chen et al., 2018). Previously, we revealed MAN as a good anti-rheumatic drug candidate. It efficiently protected joints and eased inflammatory manifestations in rats with collagen-induced arthritis (CIA) and adjuvant-induced arthritis (AIA) (Zuo et al., 2018a; Zuo et al., 2018b). Compared with the representative DMARD leflunomide, MAN treatment led to more profound decrease of VEGF in synovium. This encouraging finding implied that MAN could be more efficient in controlling angiogenesis than conventional anti-rheumatic drugs. Because angiogenesis-driven synovitis is a main cause of joint injuries, this phenomenon is especially worthy of researches. Undoubtedly, its immunoregulatory properties are essential for above therapeutic outcomes (Zuo et al., 2018b; Yin et al., 2020; Hu et al., 2021). But the altered energy metabolism could be similarly important (Yang et al., 2019; Hu et al., 2021). As the direct and most important source of energy supply in mammals, glycometabolism is sensitive to therapies. Taken the close relationship between glycolysis and hypoxia into consideration, we assumed that MAN-altered glycometabolism could down-regulate HIF-1α-governed angiogenesis in rheumatic subjects, which was basically validated by the current study.

## Materials and Methods

### Chemicals and Reagents

Immunization grade *Bacillus* Calmette-Guérin (BCG) and incomplete Freund’s Adjuvant (IFA) were supplied by Rebio Scientific (Shanghai, China). Interleukin-6 (IL-6), transforming growth factor beta (TGF-β), cyclooxygenase-2 (COX-2), angiopoietin-1 (ANG-1), laminin (LN), HIF-1α and VEGF ELISA kits were the products of MultiScience (Hangzhou, Zhejiang, China). Colorimetric kits used in the determination of concentrations of malondialdehyde (MDA), reduced glutathione (GSH), glucose, lactate, pyruvate and citric acid and catalytic activities of lactate dehydrogenase (LDH), superoxide dismutase (SOD) and NADH oxidase (NOX) were provided by either Solarbio (Beijing, China) or Keygen Biotech (Nanjing, Jiangsu, China). Lipopolysaccharide (LPS), 3-(4,5-dimethyl-2-thiazolyl)-2,5-diphenyl-2-H-tetrazolium bromide (MTT), rat basic fibroblast growth factor (bFGF) and human VEGF were purchased from Keygen Biotech (Nanjing, Jiangsu, China). Matrigel, RPMI 1640 medium, phosphate buffered saline (PBS) and fetal bovine serum (FBS) were bought from Thermo Fisher Scientific (Rockford, IL, United States). Mass spectrometry grade solvents/chemicals including methanol, acetic acid and sodium hydroxide were obtained from CNW Technologies (Shanghai, China). MAN with the purity of 99% was procured from BCYK Biotech (Nanjing, Jiangsu, China). Compound 3K (CK, a selective M2-type pyruvate kinase (PKM2) inhibitor) and stiripentol (STI, a LDH inhibitor) were purchased from Selleck Chemicals (Houston, TX, United States).

### Establishment of AIA in Rats and Treatment

All animal-involved experimental procedures were strictly in accordance with the National Institutes of Health Guide for the Care and Use of Laboratory Animals (NIH Publications No. 8023, revised at 1978). Relevant approval was obtained from the Ethics Committee of Wannan Medical College (LLSC-2020-138). Male wistar rats of 7 weeks old were used, which were supplied by Tianqin Biotechnology Co., Ltd. (Changsha, Hunan, China) and housed in a specific-pathogen free laboratory. We have systematically evaluated anti-rheumatic effects of MAN using both AIA and CIA rats, and leflunomide and methotrexate were adopted as positive drugs in these researches (Zuo et al., 2018a; Zuo et al., 2018b). This study aimed to investigate its anti-angiogenesis mechanism rather than therapeutic efficacies, and therefore no control reagent was included. Experimental animals were divided into three groups randomly (with six rats each) after a week accommodation: normal healthy control, AIA model control and MAN-treated group. Before the immunization, BCG was finely grinded in IFA to prepared complete Freund’s adjuvant (CFA, containing 20 mg/ml of BCG) using a glass mortar. On day 0, AIA models and MAN-treated AIA rats were given an intradermal injection of 0.1 ml CFA at planar of the left hind paw. Since day 1, rats in MAN treatment group were orally treated by MAN (in the form of micro-emulsion) for 32 days, and the other two groups were given 0.5% sodium carboxymethyl cellulose solution in parallel. The preparation of MAN micro-emulsion was according to our previous report (Xu et al., 2017). Briefly, MAN, oleic acid, isopropyl myristate, cremophor EL, and ethanol were thoroughly mixed at appropriate proportions by the aid of magnetic stirring, and the resulting product was diluted with an equal volume of water to obtain homogeneous micro-emulsion. It has been revealed that MAN can efficiently cure experiment arthritis at the dose 40–50 mg/kg/day (Zuo et al., 2018a; Zuo et al., 2018b; Yang et al., 2019; Hu et al., 2021). Due to the improve delivery efficacy, therapeutic doses of MAN in the form of micro-emulsion can be further reduced (Xu et al., 2017; Zuo et al., 2018b). Based on preliminary trials, the therapeutic dose of MAN in this study was set at 30 mg/kg/day.

### Sampling and Clinical Evaluation

During the observational period, body weights and arthritis scores of rats were periodically recorded (Zuo et al., 2018a; Zuo et al., 2018b). At day 27, 2 ml of anticoagulation blood from each rat was collected. Levels of oxidative parameters, cytokines and metabolites within these samples were determined using corresponding quantification kits. Five days later, the rats were sacrificed. Portion of the anticoagulation blood was immediately fed to a PE-6800 VIT blood cell counter (Pukang Biotech, Shenzhen, Guangdong, China) for complete blood cell count (CBC). The remaining blood was centrifugated to separate plasma, which was used in the following LC-MS analysis. Synovium from knee joints as well as spleen and thymus were collected and homogenized, which were used for the determination of VEGF. Ankle joints of the hind limbs were dissected from the body and preserved in neutral formalin buffer. Local expression of HIF-1α and VEGF were assessed by the immunohistochemical method. Briefly, the joints embedded in paraffin were sectioned and mounted on glass slides. The specimens were then treated by hydrogen peroxide, citric acid, normal serum, primary antibodies, and horseradish peroxidase (HRP)-conjugated secondary antibody in turns. After a further incubation with 3,3-diaminobenzidine, the immunized proteins were visualized, which was followed by hematoxylin-based counterstaining.

### *In vivo* Matrigel Plug Assay

To validate the effects of MAN treatment on AIA-related pathological angiogenesis *in vivo*, matrigel plugs were implanted in half rats of each group 3 days before sacrifice. Prior to this procedure, matrigel was thawed overnight in a refrigerator under 4°C, and mixed with bFGF at a concentration of 150 ng/ml. A subcutaneous injection with 0.5 ml of this mixture was performed on abdomen. The next day, every rat was manually checked to confirm whether the gel was successfully turned into solid plugs. When the rats were killed at day 32, the matrigel plugs were retrieved. Angiogenesis status in plugs from different rats was preliminarily assessed by morphological observation. Subsequently, the specimens were fixed in formalin, embedded in paraffin, and sectioned. After that, the sections were mounted on glass slides, dewaxed and rehydrated. Finally, hematoxylin/eosin (H&E) staining was carried out.

### Cell-Based Anti-Angiogenesis Assays

Human umbilical vein endothelial cells (HUVECs) were used in experiments *in vitro*, which were kindly given by Prof. Kun Lv (Wannan Medical College) and cultured under routine conditions (37°C, 5% CO_2_). In MTT assay, the cells (5 × 10^3^ cells/well) seeded in 96-wells plates were stimulated by MAN at various concentrations for 24 h. Subsequently, 10 μl of MTT solution (5 mg/ml) was added into each well. After the incubation in dark for 4 h, formed formazan was resolved in dimethyl sulfoxide, and the amounts of living cells were assessed by optical density read at 570 nm. In the following experiment, HUVECs (2 × 10^4^ cells/well) cultured in a 48-wells plate were stimulated by LPS (500 ng/ml) for 1 h, and then treated by MAN at various concentrations. Twelve hours later, VEGF within medium was determined using an ELISA kit according to the manufacturer’s protocol. Based on results from above experiments, an optimized treatment concentration with potent anti-angiogenic effects was chosen. Some other HUVECs (5 × 10^5^ cells/well) were seeded in 6-wells plates. When reaching 80% of confluence, cellular monolayer on the bottom was scratched straightly using a pipette tip. Cell debris was removed by washing. Some of the remaining cells were maintained in medium supplemented with 10% serum from RA patients or in the combination of MAN, while the controls were incubated by normal human serum-containing medium. The use of clinical samples was following the procedures and guidelines of the Declaration of Helsinki. The written informed consents were obtained from all participants. HUVECs migrated into the linear wound were observed 2 days later. In migration assay, HUVECs (1 × 10^5^ cells/well) were seeded in the upper chamber of matrigel-coated transwell with the pores of 8 μm (Chamber Matrigel Invasion 24 Well, Corning). The lower chamber was filled with complete medium supplemented with 200 ng/ml VEGF or in the combination of MAN. After 24 h incubation, the cells infiltrated through transwell were fixed in paraformaldehyde, and stained with crystal violet. In a duplicate experiment, the migrated cells were digested, and counted by the aid of a Beckman Coulter Vi-CELL XR. To assess the tube-forming ability, HUVECs (5 × 10^5^ cells/well) were cultured in a matrigel-coated 6-wells plate. The cells received the same treatments as described above. By the end of incubation, HUVECs-constructed capillary tubules were observed and photographed.

### Arterial Ring Experiment

During the sacrifice, some normal and AIA rats were immersed in 75% ethanol for 2 min. Thoracic aorta of rats was cut along the spine under a sterilized condition, which was then placed in a culture dish and extensively rinsed by sterile PBS. The connective tissues were carefully stripped off, and the blood vessels were cut into rings with the size of 1 mm high. A pre-chilled 48-wells plate was coated with matrigel (100 μl per well), and kept under 37°C for 15 min to solidify the gel. Afterwards, the arterial rings were placed on the matrigel-coated wells, and maintained in 500 μl complete medium (RPMI 1640 medium supplemented with 10% FBS). Half of the samples were stimulated by MAN at an optimized concentration. Every 2 days, the medium was replaced. At day 2 and 5, the samples were photographed. Migration and growth of arterial ring-derived endothelial cells were observed and compared.

### LC-MS Analysis of Metabolites in Plasma

Because systematic metabolic alteration will eventually reflect in changes of metabolites in blood, we performed the metabonomics study using rat plasma. A 60 μl aliquot of plasma was thoroughly mixed with 40 μl of water and 300 μl of methanol. The mixture was then subjected to supersonic treatment in an ice-water bath for 15 min. After being kept under −40°C for 1 h, the samples were centrifuged at 12,000 rpm under 4°C for 15 min. The supernatant (300 μl) was then dried using a vacuum centrifugal concentrator (Centrivap console, Labconco Company, United States). Analytes were prepared by resolving the residues in 200 μl water, which were further filtrated and injected into LC-MS apparatus for analysis. Chromatographic separation was performed on a Dionex ICS-6000 system (Thermo Scientific) equipped with a Dionex IonPac AS11-HC (2 × 250 mm) column. Sodium hydroxide solution (100 mM in water) and water were adopted as the mobile phase A and B respectively, which were mixed in a constant rate of 2:3. Before entering the mass spectrometer, base in solvent was neutralized by an additional flow of 2 mM acetic acid in methanol. Column and auto-sampler temperatures were set at 30°C and 4°C, respectively. Separated metabolites were then detected by a 6500 QTRAP^+^ triple quadrupole mass spectrometer (AB Sciex) coupled with an ESI interface under MRM mode. Some key analytical parameters were summarized as below: spray voltage, −4500 V; ionization temperature, 450°C; ion source gas 1, 45 psi; ion source gas 2, 45 psi; curtain gas, 30 psi. Before the analysis of samples, calibration curves of all the metabolites were developed using standard solutions under the same conditions. The parent/daughter ions together with the data about linear correlation, quantification range, precision and accuracy were included in Supplementary Table S1.

### Evaluation of Metabolism-Mediated Anti-Angiogenic Effects of MAN *in vitro*

HUVECs (5 × 10^5^ cells/well) were seeded in 6-wells plates beforehand. After attachment, the cells were treated with MAN at various concentrations for 24 h. Thereafter, the medium was collected, and levels of glucose, LDH and SOD within were detected using corresponding kits. Based on the results, an optimal concentration of MAN with good metabolism regulatory potentials was chosen. Subsequently, some other HUVECs were challenged by LPS (500 ng/ml), and incubated with MAN-containing medium for 24 h. Levels of glucose, LDH and SOD in the medium were determined likewise. Some representative cytokines involved in angiogenesis including IL-6, ANG-1, TGF-β, LN, and COX-2 were quantified too. According to the results revealed by metabonomics analysis, possible regulatory mechanism of MAN on glucose metabolism was proposed. In this assay, certain metabolic inhibitors were used as reference reagents to treat LPS-primed HUVECs alone or in the combination of MAN. Some metabolites as well as HIF-1α and VEGF in medium collected from the culture system were quantified using appropriate kits. With the same experimental arrangement, the tube-forming assay was carried out.

### Statistical Analysis

All the data were expressed as mean ± standard deviation. Six data were included in most analyses involved in animal specimens, and all the *in vitro* experiments were performed in triplicates. Based on the one-way analysis of variance coupled with Tukey post hoc test, statistical differences were considered as significant when *p* values <0.05 or 0.01. All the statistical analyses were performed by the aid of GraphPad Prism 8.0 (Cary, NC, United States).

## Results

### MAN Exhibited Anti-Angiogenesis Potentials in AIA Rats

**FIGURE 1:**
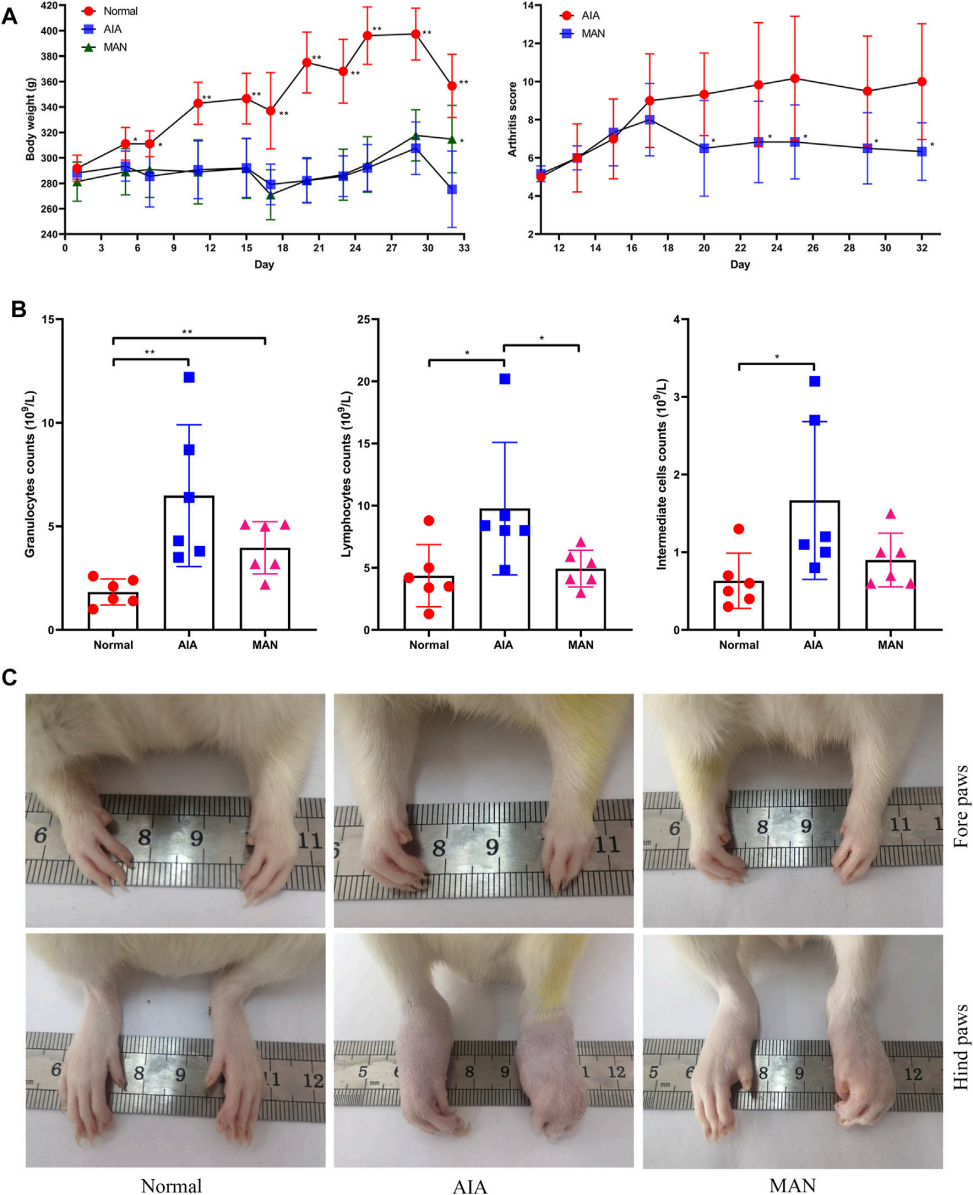
MAN alleviated severity of AIA in rats after continuous oral treatment. **(A)**, periodic changes of arthritis scores and body weight of AIA rats (*n* = 6); **(B)**, CBC analysis of anticoagulation blood obtained upon the sacrifice of rats (*n* = 6); **(C)**, morphological observation of paws by the end of experimental period. Statistical significances in image A: ^*^ *p* < 0.05 and ^**^ *p* < 0.01 compared with AIA model controls.

Although MAN treatment did not restore body weight gain in AIA rats, it caused significantly decrease in arthritis score since day 20 (Figure 1A). The increase in counts of granulocytes, lymphocytes and intermediate cells in peripheral blood indicated the systematic inflammation in AIA rats (Figure 1B). This abnormality was efficiently restored by MAN therapy. In fact, there was no obvious difference about lymphocyte and intermediate cell counts between MAN-treated AIA rats and normal healthy rats. Anti-inflammatory effects of MAN eventually led to improved arthritic severity, indicated by reduced paw swelling and joints deformation (Figure 1C).

**FIGURE 2:**
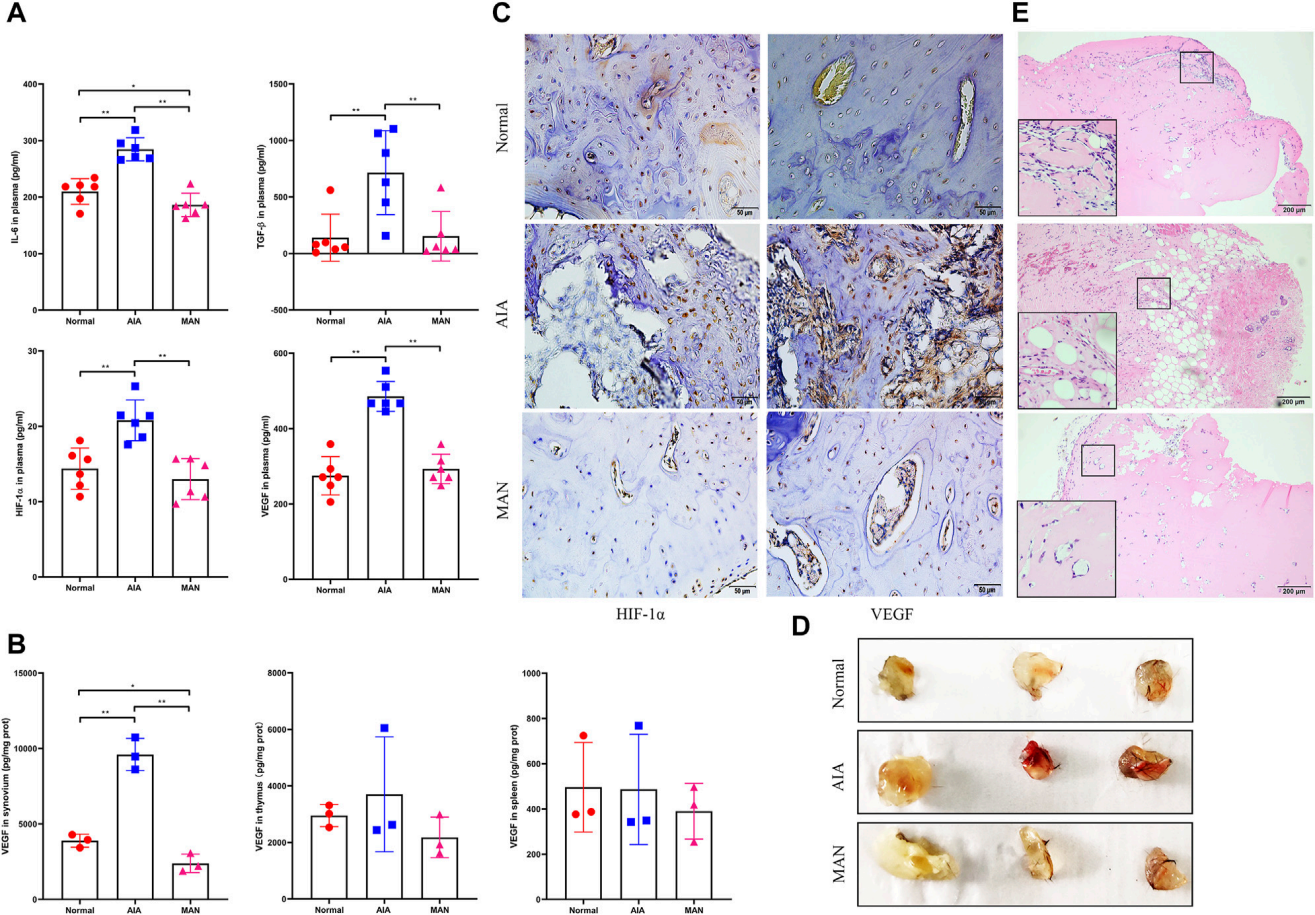
MAN inhibited pathological angiogenesis in AIA rats. **(A)**, levels of angiogenic cytokines in plasma (*n* = 6); **(B)**, levels of VEGF in different tissues of rats (*n* = 3); **(C)**, local expression of HIF-1α and VEGF in cartilage of joints; **(D)**, morphological observation of matrigel plugs implanted in different rats; **(E)**, H&E staining-based histological examination of above matrigel plugs.

Blood parameters are important and mostly used diagnostic indicators for most diseases, including angiogenesis-involved rheumatic diseases (Connolly et al., 2010). We preliminarily evaluated the possible anti-angiogenic effects of MAN on AIA rats by analyzing some cytokines in blood. Unfortunately, levels of ANG-1, COX-2 and LN were too low to be detected. Fluctuation of IL-6, TGF-β, HIF-1α and VEGF was consistent to our speculation. All of them were significantly increased in AIA rats. After MAN treatment, they were reduced to basal levels (Figure 2A **)**. We wondered if this effect was tissue specific, and therefore determined VEGF in synovium and immune organs, two key presumed targets of MAN treatment in rheumatic rats (Zuo et al., 2018a; Zuo et al., 2018b). It was revealed that MAN generally reduced VEGF production in these tissues, while the change in synovium was especially significant (Figure 2B). The immunohistochemical examination showed that by the degradation of cartilage, overexpression of HIF-1α and VEGF occurred in AIA rats, and this situation was attenuated in MAN-treated AIA rats (Figure 2C).

Preliminary trials found that the matrigel implanted in AIA rats was largely absorbed after 4 days. Hence, we retrieved matrigel plugs 3 days after implantation. As shown in Figure 2D, the plugs from AIA models exhibited darker red color than those collected from normal healthy and MAN-treated AIA rats. H&E staining-based histological examination demonstrated that under AIA conditions, notable blood cells infiltration as well as pannus formation occurred in matrigel plugs. MAN treatment was obviously unfavorable for neovascularization *in vivo*, and no vascular structure was found in matrigel plugs retrieved from MAN-treated AIA rats (Figure 2E).

### MAN Suppressed Angiogenesis *in vitro*

**FIGURE 3:**
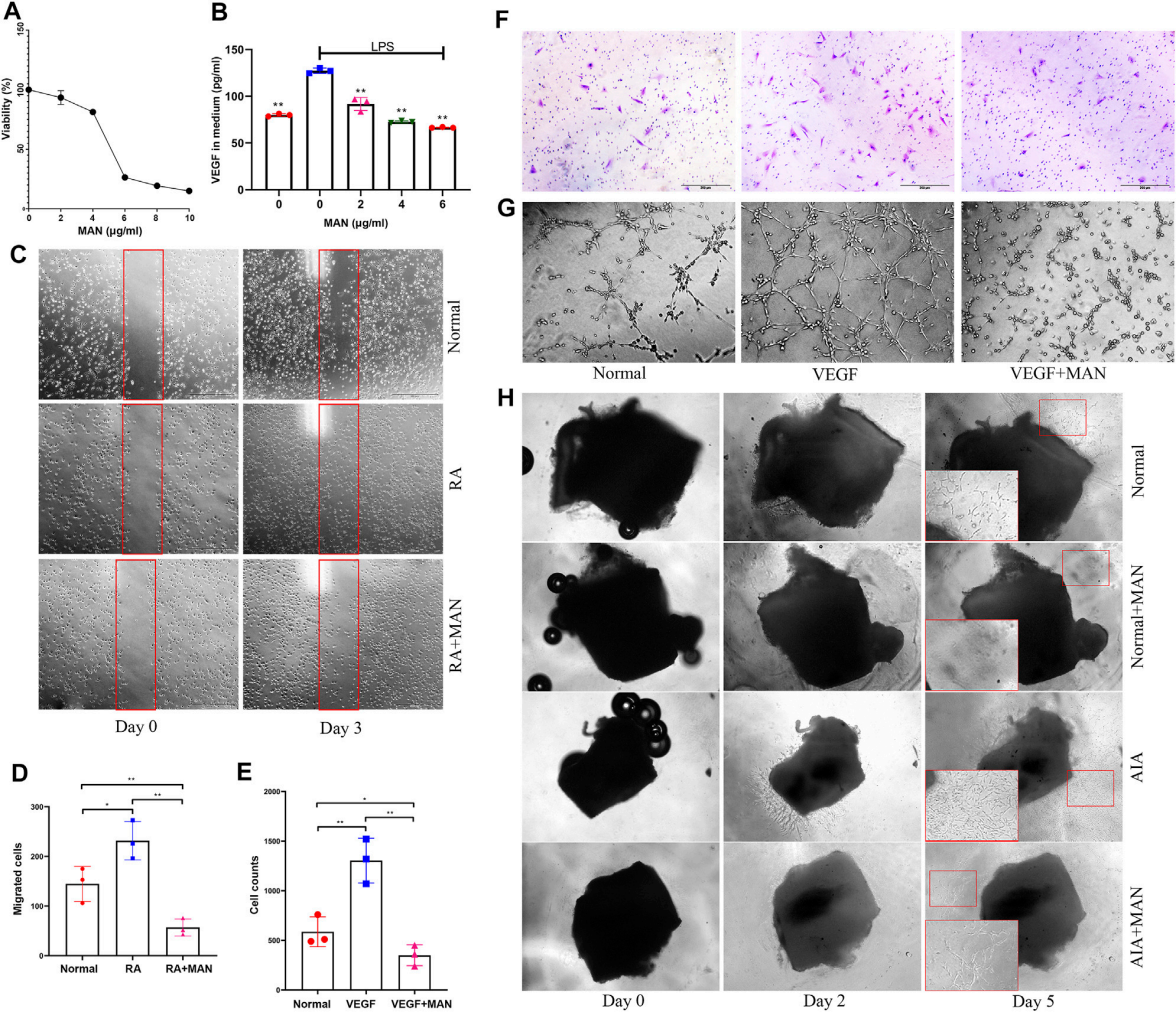
MAN inhibited angiogenesis potentials of endothelial cells *in vitro*. **(A)**, the effects of MAN on HUVECs viability, assessed by MTT assay (*n* = 6); **(B)**, MAN inhibited VEGF production in LPS-primed HUVECs in a concentration-dependent manner (*n* = 3); **(C)**, MAN inhibited the migration of RA serum-cultured HUVECs in scratch assay; **(D)**, the quantification result of assay C; **(E)**, MAN decreased the counts of HUVECs infiltrated through transwell in the existence of VEGF; **(F)**, the infiltrated HUVECs was dyed with crystal violet in the replicate of assay E; **(G)**, MAN impaired the capacity of VEGF-primed HUVECs to form tubes *in vitro*; **(H)**, suppressive effects of MAN on migration/growth of rat arterial ring-derived endothelial cells under both normal and AIA conditions. Statistical significances in image B: ^**^ *p* < 0.01 compared with LPS-primed cells.

MAN stimulus did not significantly affect the viability of HUVECs at 4 μg/ml and below (Figure 3A). Next, we treated LPS-primed HUVECs with MAN at 2, 4, and 6 μg/ml. As illustrated in Figure 3B, MAN attenuated LPS-induced increase in VEGF production in a concentration-dependent manner in this range, and the significant effect can be achieved at the concentration as low as 2 μg/ml. To rule out cytotoxicity-brought interferences, 4 μg/ml was finally adopted as the optimal treatment concentration in the following assays. In wound healing assay, we found that RA serum significantly promoted HUVECs migration (Figure 3C). MAN totally abrogated this trend. In fact, the cells migrated into the wound in the presence of MAN were even less than untreated controls (Figure 3D). Cell count results of the transwell assay similarly concluded that MAN antagonized VEGF-induced migration of HUVECs (Figure 3E). By the aid of crystal violet staining, optical microscope observation obtained the same conclusion in the replicate experiment (Figure 3F). Furthermore, MAN impaired the tube-forming ability of VEGF-primed cells (Figure 3G). In arterial ring experiment, endothelial cells migrated from vessels onto matrigel-coated wells, and formed capillary sprouts after being cultured *in vitro* for 2 days. Compared with normal controls, migration and growth of AIA rat arterial ring-derived endothelial cells were much faster. MAN exhibited potent inhibitory effects on these phenomena (Figure 3H). Overall evidences solidly confirm that MAN is effective against RA-related pathological angiogenesis.

### MAN Inhibited Aerobic Glycolysis in AIA Rats

**FIGURE 4:**
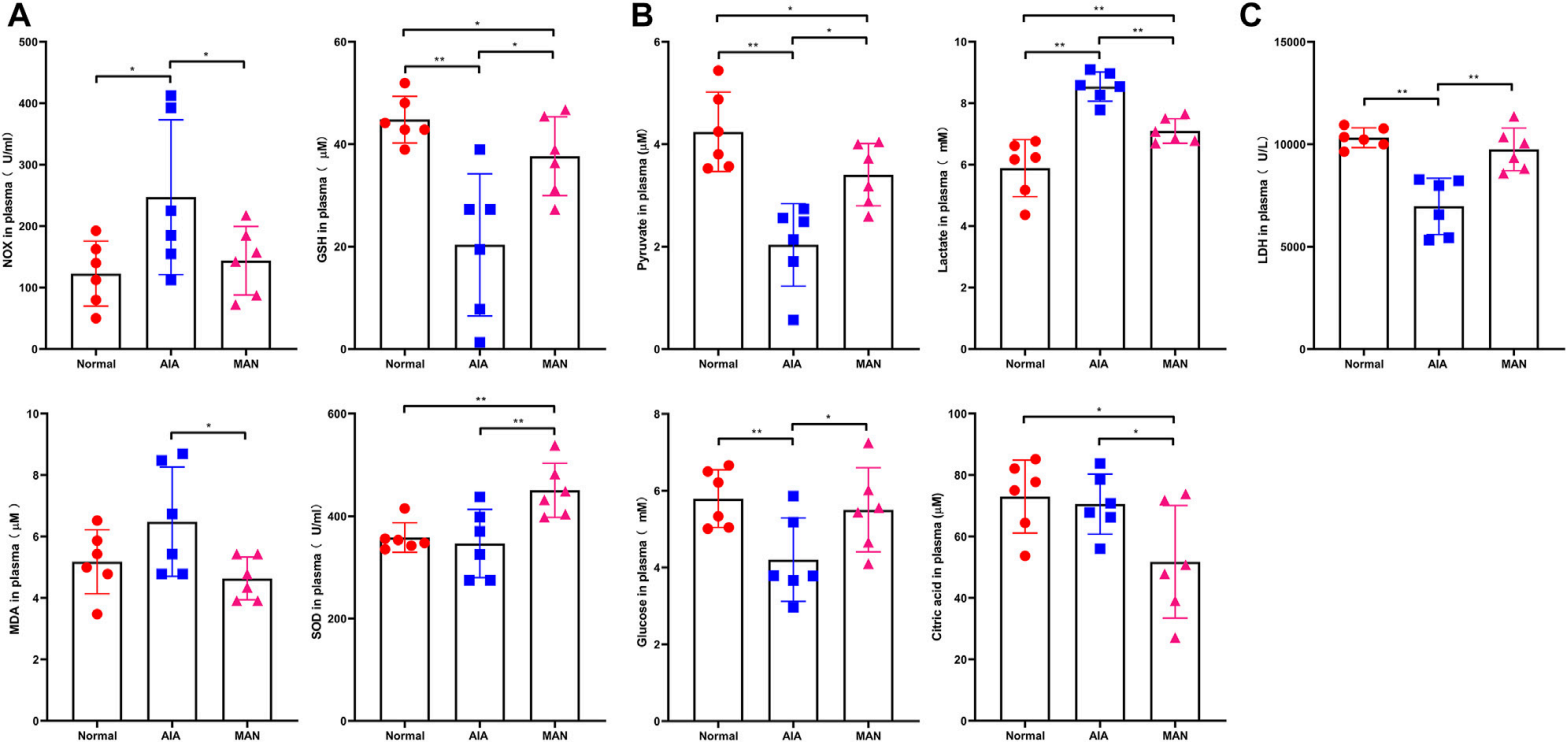
MAN restored oxidative stress and glycometabolism alteration in AIA rats. **(A)**, levels of NOX, MDA, GSH and SOD in plasma (*n* = 6), and all parameters in this and following assays were detected using rat blood samples collected at day 27; **(B)**, levels of glucose, pyruvate, lactate and citric acid in plasma (*n* = 6); **(C)**, catalytic activity of LDH in the rat plasma (assessed by the production of pyruvate *ex vivo*, *n* = 6).

Because energy metabolism disturbance is always accompanied with oxidative stress changes, we firstly determined levels of NOX, GSH, MDA and SOD in plasma (Eltzschig and Carmeliet, 2011). In AIA rats, it was observed that levels of NOX and MDA were increased, while GSH was decreased. Meanwhile, SOD was unchanged. These facts demonstrated that reactive oxygen species (ROS) scavenging system in AIA rats was not crippled, and the high oxidative stress observed could be largely caused by altered energy metabolism. MAN significantly restored all the altered oxidative parameters and promoted the activity of SOD in AIA rats (Figure 4A). It is known that mitochondria are the major intracellular source of ROS, i.e., dysfunction of mitochondria impairs aerobic oxidation and promotes ROS production simultaneously (Ott et al., 2007). Therefore, the recovery effects of MAN on above parameters do not only validate its anti-oxidative potentials, but also indicate the altered energy metabolism status in treated rats.

We found blood glucose was reduced in AIA rats, indicating the increased glucose consumption. However, it did not lead to pyruvate accumulation. To the opposite, levels of pyruvate were decreased. Two possibilities were proposed to explain the abnormal depletion of pyruvate under this situation: 1, aerobic oxidation velocity was even further promoted; 2, increasing pyruvate was converted to lactate. Changes of tricarboxylic acid cycle (TCA) intermediates and lactate were the key to clarify these hypotheses. We observed obviously higher levels of lactate in AIA rats, while citric acid was not increased (Figure 4B). It suggests that above phenomenon was mainly caused by up-regulated aerobic glycolysis. MAN treatment restored all these changes in general. As aerobic glycolysis is characterized by the accelerated production of lactate, we subsequently detected activity of LDH, which controls the conversion of lactate and pyruvate. As speculated, its capacity producing pyruvate was impaired in AIA rats, which was recovered by MAN (Figure 4C). Even though we did not understand how MAN reshaped glycometabolism in AIA rats in details, above facts demonstrate that MAN regimen suppressed aerobic glycolysis, a typical pro-inflammatory metabolic phenotype (Eltzschig and Carmeliet, 2011). MAN-induced metabolic changes thereby could contribute a lot to its therapeutic effects on AIA by easing inflammation.

### MAN Reshaped Glycometabolism of AIA Rats as a Whole

**FIGURE 5:**
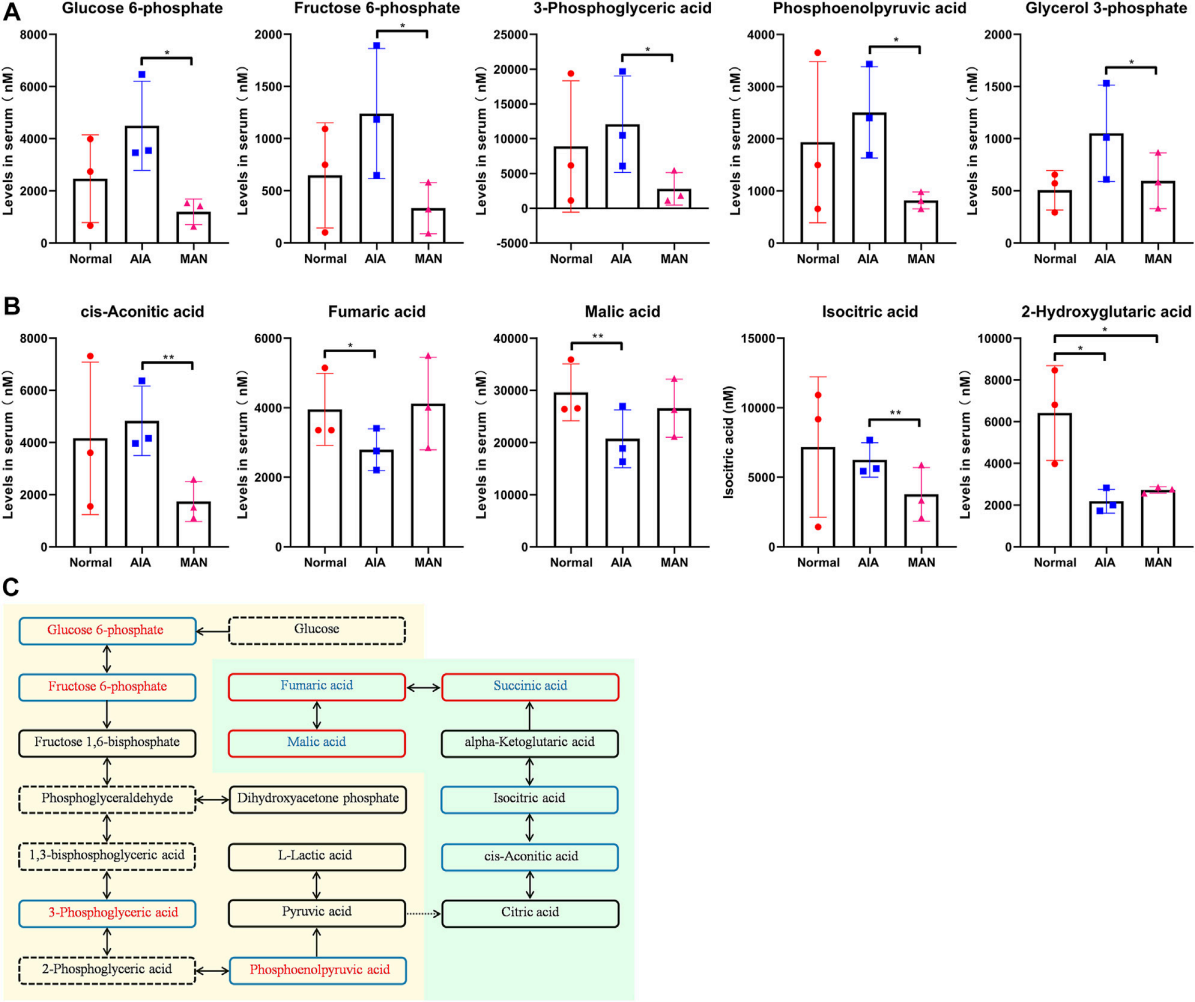
MAN rebalanced glycolysis and aerobic oxidation in AIA rats. **(A)**, levels of glycolysis-related differentiating metabolites among groups (*n* = 3), and all the metabolites shown in this and following images were detected by LC-MS in plasma obtained upon the sacrifice of rats; **(B)**, levels of aerobic oxidation-related differentiating metabolites among groups (*n* = 3); **(C)**, changes of metabolic flow occurred in AIA and MAN-treated AIA rats (dotted line indicated undetected compounds, colors of the frame indicated MAN-brought changes, colors of the text indicated AIA-related changes, blue and red colors indicated down-regulation and up-regulation respectively).

To better characterize MAN treatment-induced metabolic consequences, we accurately quantified 56 glycometabolism-related metabolites in rat plasma. Raw total ion chromatograms and quantification results were included in Supplementary Tables S2, S3, respectively. The metabolites with significant differences among groups were discussed below. Many glycolysis-related metabolites including glucose-6-phosphate, fructose-6-phosphate, 3-phosphoglyceric acid and phosphoenolpyruvic acid were increased in AIA rats (Figure 5A). Similar changes happened to glycerol-3-phosphate (a derivative of dihydroxyacetone phosphate). MAN treatment significantly decreased all these metabolites. To the opposite, TCA intermediates fumaric acid and malic acid were greatly reduced under AIA conditions, which were restored by MAN (Figure 5B). As a derivative of α-ketoglutaric acid, 2-hydroxyglutaric acid fluctuated in the similar pattern to the two metabolites mentioned above. Confusingly, MAN treatment reduced but not increased levels of isocitric acid and *cis*-aconitic acid in AIA rats, another two key metabolites involved in aerobic oxidation. To better display the changes on metabolic flow, we mapped all the metabolites with changes above 1.2 fold (irrespective of statistical significance) into Figure 5C. In this figure, undetected compounds were annotated by dotted line. Colors of the frame and text indicated MAN-brought and AIA-related changes, respectively. Blue and red colors indicated down-regulation and up-regulation, respectively. It demonstrates that MAN systematically inhibited the abnormally up-regulated glycolysis in AIA rats, and provides useful clues to elucidate the inconsistent effects of MAN on TCA intermediates. α-Ketoglutaric acid was revealed as the turning point in the regulation of MAN on aerobic oxidation. It is reasonable to assume that MAN promoted succinic acid and its downstream metabolites synthesis, which led to depletion of the upstream substrates. This speculation is plausible, as α-ketoglutarate dehydrogenase (KGDH, the enzyme accounts for succinic acid synthesis) is sensitive to inflammation. Upon LPS stimulus, its expression is significantly decreased (Ji et al., 2020). As an anti-inflammatory reagent, MAN could restore the function of this enzyme.

**FIGURE 6:**
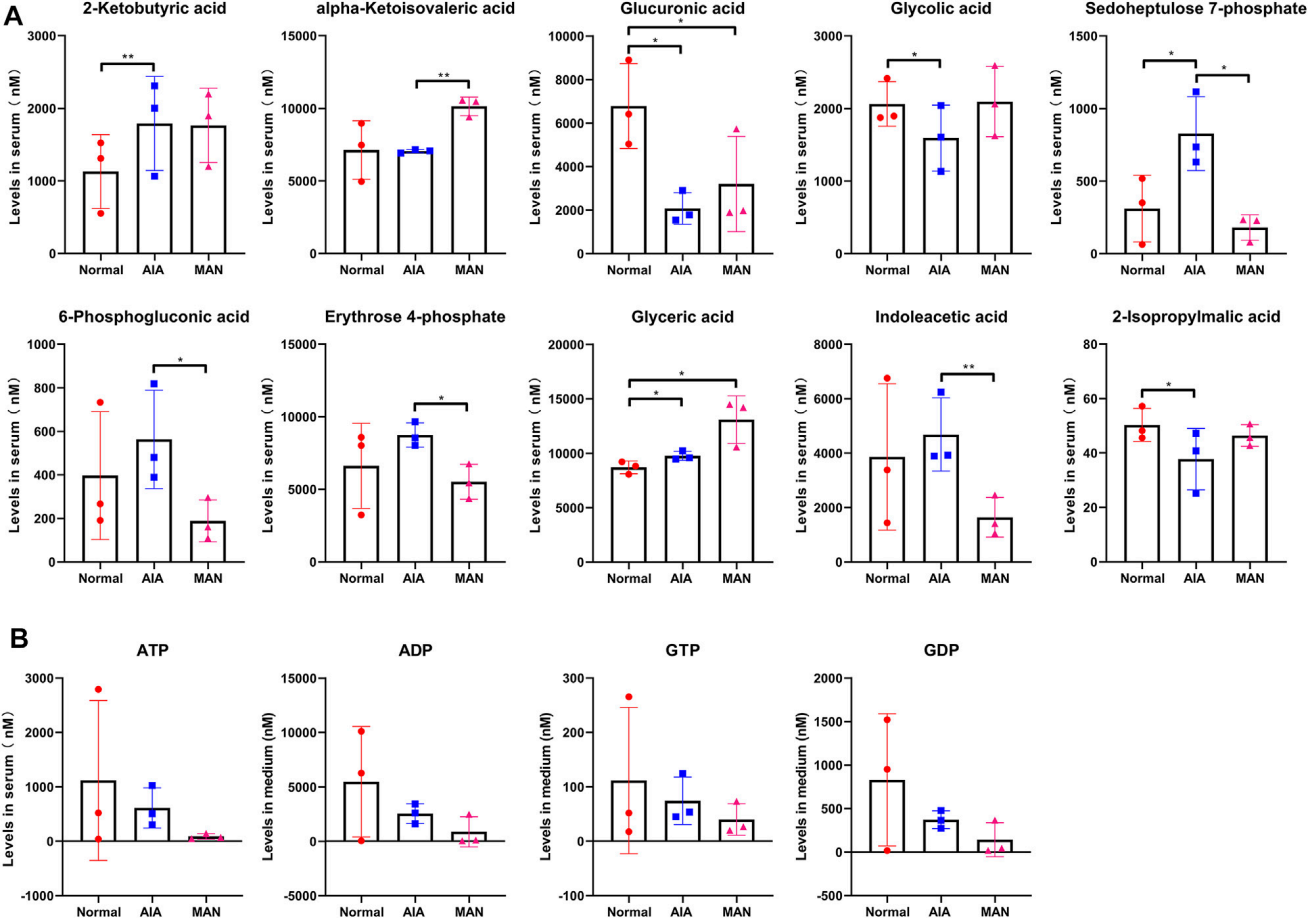
Supporting evidences for the regulation of MAN on glucose metabolism in AIA rats. **(A)**, levels of glycometabolism metabolites indirectly related to glycolysis and aerobic oxidation of glucose detected in rat plasma (*n* = 3); **(B)**, levels of high-energy phosphate compound in rat plasma (*n* = 3).

Some other differentiating metabolites are displayed in Figure 6. These data support the idea that MAN treatment rebalanced glycometabolism in AIA rats as a whole. Changes of 2-isopropylmalic acid, glycolic acid, α-ketoisovaleric acid and glyceric acid further prove that MAN treatment facilitated aerobic oxidation. Increased levels of 2-ketobutyric acid and indoleacetic acid indicate the accelerated amino acids degradation in AIA rats, which favors the replenishment of carbohydrate pool. Changes in 6-phosphogluconic acid, glucuronic acid, erythrose 4-phosphate and sedoheptulose 7-phosphate basically suggest that MAN down-regulated pentose phosphate pathway (PPP) in AIA rats, and affected phosphogluconate pathway and glucuronate pathway (Figure 6A). There was no obvious difference concerning levels of ADP, ATP, GDP and GTP among these groups (Figure 6B). It was mainly caused by huge individual variations. Because of the limited sample size, some meaningful changes could be covered up by individual differences. For example, the changes on lactate revealed previously were not observed in this LC-MS analysis. Nonetheless, it can be noticed both GTP and ATP (two typical high-energy substances) were decreased by MAN, which solidly confirms that this treatment curbed energy generation in AIA rats. As for ATP, we further investigated its changes in following *in vitro* experiments.

### Inhibition of MAN on Aerobic Glycolysis Contributed to the Anti-Angiogenesis Effects

**FIGURE 7:**
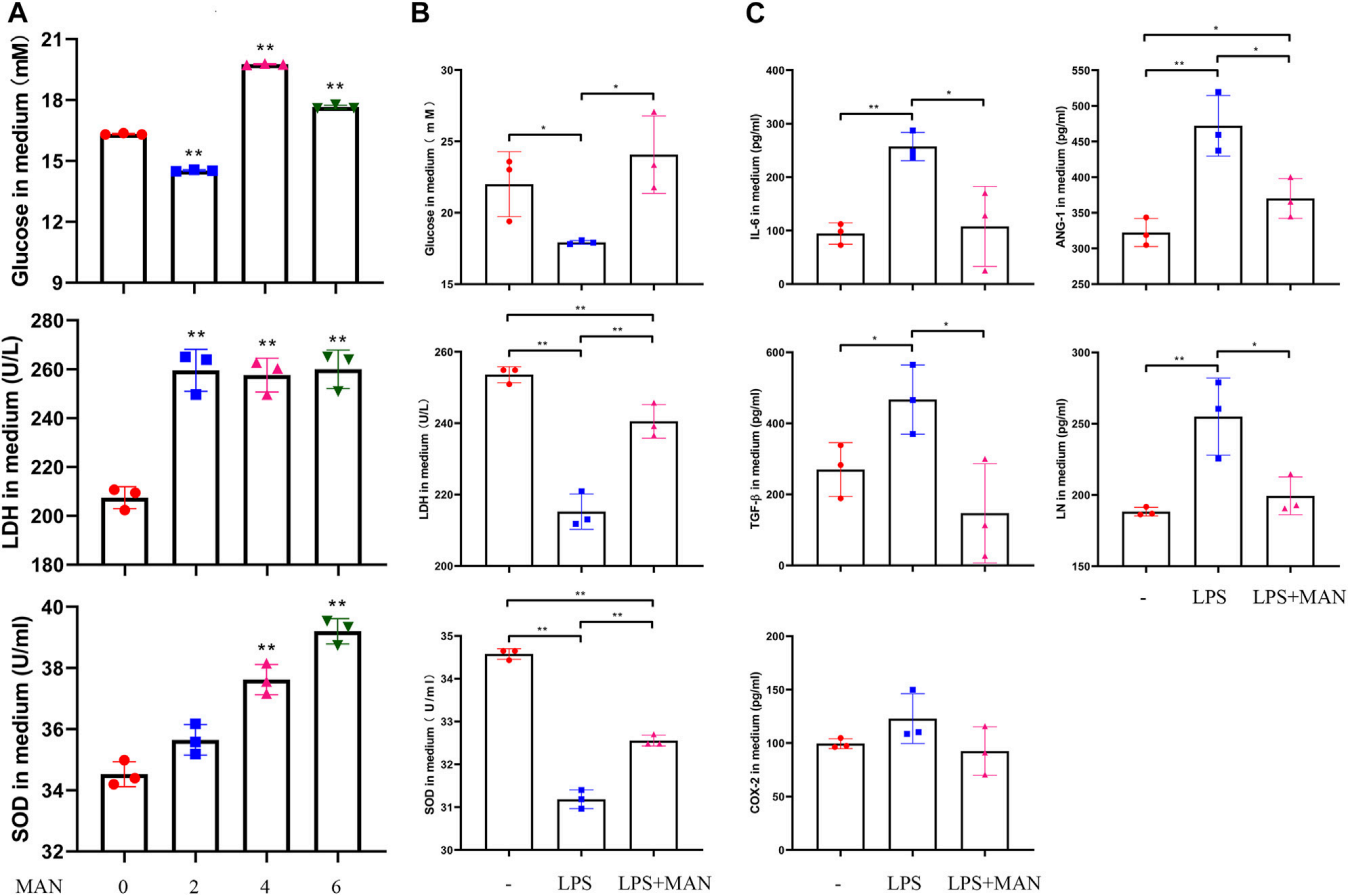
MAN affected glucose oxidation of HUVECs *in vitro*. **(A)**, levels of glucose, LDH and SOD in culture medium from the cells receiving MAN treatments at various concentrations (*n* = 3); **(B)**, levels of glucose, LDH and SOD in culture medium from the cells treated by LPS or in the combination of MAN (*n* = 3); **(C)**, levels of angiogenic cytokines released by cells from assay B. Statistical significances in image A: ^**^ *p* < 0.01 compared with untreated cells.

We firstly treated HUVECs with MAN at various concentrations, and subsequently analyzed some metabolic indicators in culture medium. It was observed that MAN at 4 μg/ml curbed the consumption of glucose most effectively, while its inhibition on the pyruvate-synthesizing capacity of LDH can be achieved even under 2 μg/ml (Figure 7A). Meanwhile, MAN promoted SOD activity in a concentration-dependent manner. Collectively, it suggested that 4 μg/ml could be an optimal concentration for MAN to exert metabolism regulatory effects on inflammatory cells. LPS-induced inflammation caused similar changes on HUVECs to those observed in AIA rats, which were then restored by MAN (Figure 7B). Besides its effects on VEGF production shown previously, MAN inhibited LPS-induced release of many other angiogenic cytokines with high efficiencies, including IL-6, ANG-1, TGF-β, LN, and COX-2 (Figure 7C).

**FIGURE 8:**
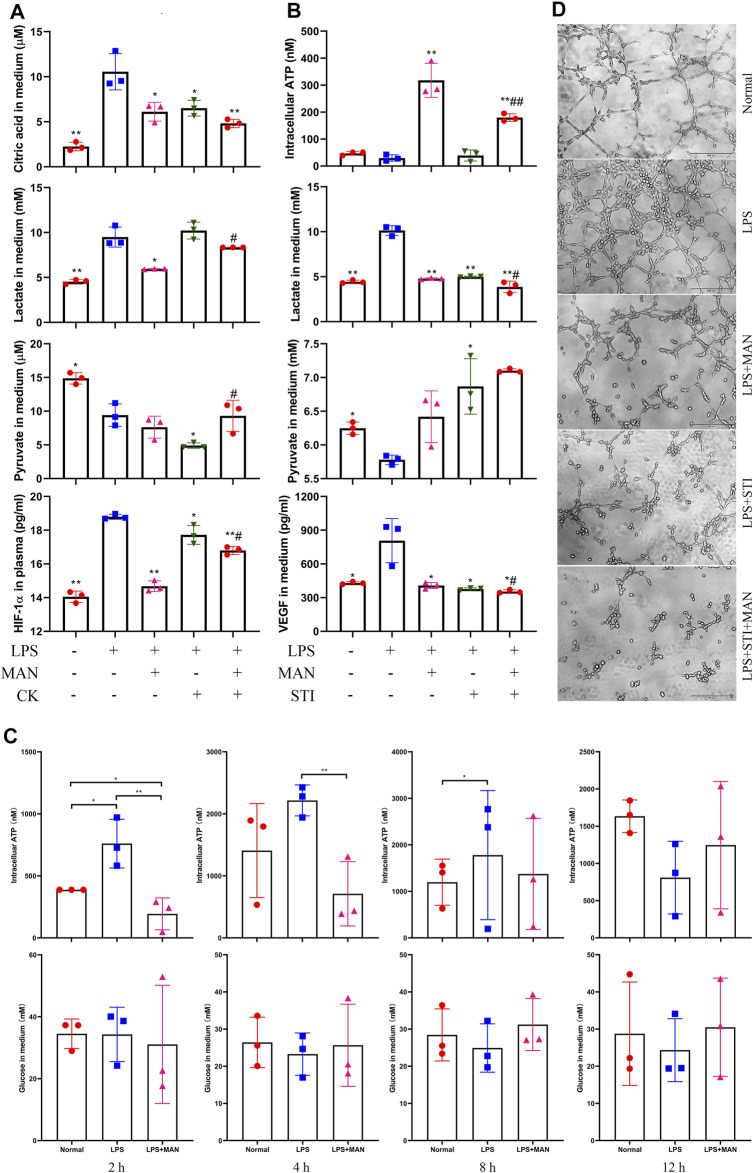
Inhibition of MAN on inflammation-caused aerobic glycolysis contributed to its anti-angiogenesis properties. **(A)**, levels of citric acid, lactate, pyruvate and HIF-1α in culture medium from LPS-primed HUVECs receiving PKM2 inhibitor CK or/plus MAN stimulus (*n* = 3); **(B)**, levels of lactate, pyruvate and VEGF in culture medium as well as intracellular ATP of LPS-primed HUVECs receiving LDH inhibitor STI or/plus MAN stimulus (*n* = 3); **(C)**, dynamic changes of intracellular ATP and extracellular glucose in HUVECs culture system in the presence of LPS or LPS + MAN (*n* = 3); D, STI and MAN exerted similar and synergistic effects on tube-forming capability of LPS-primed HUVECs *in vitro*. Statistical significance in image A and B: ^*^ *p* < 0.05 and ^**^ *p* < 0.01 compared with LPS-primed cells; ^#^ *p* < 0.05 and ^##^ *p* < 0.01 compared with cells receiving LPS + MAN treatment.

Pyruvate is the end product of glycolysis and the direct substrate of TCA. Thereby, pyruvate kinase controls a key rate-limiting step of glucose oxidation, and inhibition on this enzyme will effectively hamper glycolysis. To test if this mechanism was involved in MAN-induced metabolism alteration, we investigated the effects of CK on LPS-primed HUVECs. Although both CK and MAN decreased citric acid production, CK showed different effects to MAN concerning the changes of pyruvate and lactate. CK decreased HIF-1α levels, while MAN-brought changes were more profound (Figure 8A). Meanwhile, CK partially antagonized the inhibition of MAN on HIF-1α secretion. Overall evidences suggest that down-regulation of glycolysis itself cannot mimic the effects of MAN on AIA. In the replicate experiment, CK was replaced by STI, considering LDH is a switch of glucose metabolism by converting pyruvate and lactate, two key variables determining aerobic glycolysis status. In this assay, we tested VEGF instead of HIF-1α. MAN and STI exerted similar effects on VEGF, pyruvate and lactate, and combined use of them achieved obvious synergistic effects (Figure 8B). It basically confirms that MAN intervened into aerobic glycolysis through a similar mechanism with STI. Interestingly, compared with untreated controls, LPS stimulus did not increase ATP production in HUVECs after 24 h culture, but achieved an opposite outcome, although the difference was not significant. Different from STI, MAN increased intracellular ATP in LPS-stimulated HUVECs. Because inflammation is always accompanied with accelerated energy metabolism, these observations were confusing. We assumed that it was caused by the changes on available glucose. After a long time culture, cells may be unable to generate enough ATP in the absence of glucose replenishment approaches. That is, more glucose consumed initially, less ATP can be produced in later stages. This theory was validated by the subsequent observation (Figure 8C). Indeed, levels of intracellular ATP in LPS-stimulated HUVECs were obviously higher than untreated cells during the first 8 h, when MAN significantly suppressed ATP production. But this situation was reversed after 12 h culture. Available glucose in medium fluctuated in the opposite pattern to ATP, despite the differences among groups were not statistically significant. In the tube-forming assay, we observed synergistic effects of MAN and STI once again (Figure 8D). Under the stimulus of LPS, *in vitro* cultured HUVECs formed intensive tubules, while their capacity was significantly impaired by either MAN or STI. When stimulated by MAN plus STI, no tubes can be formed.

## Discussion

Besides immune factors, metabolism disturbance is also implicated in RA (Mateen et al., 2016). Incidence of obesity and insulin-resistance in RA patients is higher than general population, and cardiovascular disease has been identified as the leading risk of RA-related pre-mature death (Gremese and Ferraccioli, 2011). Undoubtedly, dysregulation of immune system contributes to the metabolic complications, while the altered energy metabolism will exacerbate immune abnormalities in return. It has been confirmed that metabolism reprogramming is essential for the differentiation of immune cells (Wada, 2017). Taking glycometabolism as an example, glycolysis is always accelerated in inflammatory cells, because inflammation is a highly energy-consuming event, and glycolysis can generate ATP very fast (Zhao et al., 2020). Many glycolytic genes like hexokinase 2, phosphoglycerate kinase 1 and PKM2 were dysregulated in RA patients (Zhao et al., 2016; Bustamante et al., 2018; Xu et al., 2019). The situation reflects the metabolic and inflammatory adaptation under RA conditions. Meanwhile, it is deeply involved in pathological angiogenesis. Due to the increased recruitment of inflammatory immune cells, the inflamed synovium demands more oxygen supply. Consequently, high density vessels are constructed. But the hypoxic condition cannot be thoroughly addressed (Szekanecz et al., 2009; Leblond et al., 2017). As a result of neovascularization, more immune cells emigrate into synovium from bloodstream. The sustained relative low oxygen supply requires the resident cells to adapt to glycolytic metabolism, which further deteriorates inflammation and reinforces hypoxia-driven angiogenesis (Ivashkiv, 2020). In short, glycolysis-driven inflammation causes local hypoxia and elicits the consequent angiogenesis. It should be aware that inflammation-related hypoxic situation occurs in local tissues under the context of sufficient overall oxygen supply. Thereby, relevant metabolic alteration is termed as aerobic glycolysis (Zhao et al., 2020). The current study further confirms the abnormally up-regulated aerobic glycolysis in AIA rats (Figures 4, 5). Through above mechanism, AIA-related glycometabolism changes caused significant increase in angiogenic cytokines release (Figure 7). Based on these clues, we can conclude that appropriate metabolic interventions would be an effective way to treat AIA/RA-related angiogenesis, and consequently eased arthritic manifestations.

Local hypoxia is the common inducer of aerobic glycolysis and angiogenesis (Eltzschig and Carmeliet, 2011). Therefore, HIF-1α could be a key to elucidate the pathological metabolism-angiogenesis feedback. We analysed some representative angiogenic cytokines in rats. Levels of circulating ANG-1, COX-2, and LN can hardly be determined when AIA was spontaneously eased. Although obvious difference about levels of IL-6 and TGF-β was observed among groups, they cannot challenge the dominant role of HIF-1α in angiogenesis. On one hand, as versatile cytokines, IL-6 and TGF-β do not exclusively respond to angiogenesis. On the other hand, their transcriptional expression is controlled by HIF-1α (Wang et al., 2013). Under hypoxic conditions, HIF-1α is stabilized and translocated to the nucleus. There, it initiates the transcription of many hypoxic adaptation-related genes, including VEGF and glycolytic regulators (Ivashkiv, 2020). Targeting HIF-1α thereby will attenuate the concomitant up-regulation of angiogenesis and glycolysis. Because of this, the down-regulated HIF-1α serves as a piece of solid evidence supporting the anti-angiogenic potentials of MAN (Figures 2, 8). But fundamentally, it reflects the improved local hypoxia. It is known that hypoxia-induced mitochondria dysfunction will lead to increased product of ROS. The eased oxidative stress in MAN-treated AIA rats reveals the improved hypoxic conditions (Figure 4). Among these oxidative indicators, changes of NOX are especially meaningful. Its expression is increased in response to hypoxia. Meanwhile, it directly account for mitochondrial ROS production and HIF-1α synthesis, and contribution to angiogenesis through many different mechanisms (Schröder et al., 2009; Yuan et al., 2010). MAN-caused down-regulation of NOX does not only indicate the eased hypoxia, but also further confirms the anti-angiogenic effects. Because of the close relationship between hypoxia and metabolism, the metabolism regulatory properties of MAN are more worthy of investigation in this regard. Available clues suggest that metabolic intervention could be the priority for MAN to improve hypoxia-related angiogenesis. We found that MAN can affect glycometabolism in HUVECs without LPS stimulus, when hypoxia was absent (Figure 7). It indicates that the metabolism regulatory properties of MAN are independent of inflammation-caused hypoxia. In line with this, when HUVECs were exposed to sufficient oxygen under normoxia conditions, their ability synthesizing HIF-1α and VEGF can be still affected by MAN stimulus (Figure 8). These facts demonstrate that the innate metabolism regulatory properties empower MAN with potential ability in curbing hypoxia-governed angiogenesis.

As for how MAN attenuated hypoxia by suppressing aerobic glycolysis, there are many possible explanations. But the most plausible is that the metabolic alteration reshaped differentiation profiles of immune cells and consequently eased local inflammation. The negative effects of MAN on inflammatory differentiation of immune cells in both CIA and AIA rats have been well investigated (Zuo et al., 2018a; Yang et al., 2019; Yin et al., 2020; Hu et al., 2021). In this study, we similarly found MAN-induced decrease in IL-6 in treated AIA rats (Figure 2). The decreased ATP production could be the fundament for the impaired inflammatory differentiation (Figure 6). It is apparent that the increased ATP production fuels inflammation, and inflammation usually requires extra aerobic glycolysis to meet energy demands. But molecular mechanism under this simple logic is very complicated, and to be thoroughly understood yet. For example, ATP can act on P2X7 receptor, and consequently form a Th17 polarizing microenvironment by promoting the secretion of IL-1, IL-6, and IL-17 (Pandolfi et al., 2016). Through metabolism-sensitive pathways like AMPK, ATP accumulation will activate many inflammatory pathways (Lyons and Roche, 2018). As an early danger signal, ATP will up-regulate NF-κB pathway by activation of Duox1 via P2YR/PLC/Ca2^+^ signaling (de Oliveira et al., 2014). Undoubtedly, the inhibition of MAN on aerobic glycolysis contributed significantly to the decreased ATP production. Similar to STI, MAN may also act as a LDH inhibitor (Figure 8). But according to the results shown in Figure 5C, LDH is not the only glycolic regulator targeted by MAN, as many glycolysis-related metabolites were decreased in MAN-treated AIA rats in addition to lactate. Previously, we found that MAN selectively down-regulated nicotinamide phosphoribosyltransferase (NAMPT) signaling in CIA rats (Yang et al., 2019). NAMPT is required by glycolysis, as it potentiates the catalytic activity of glyceraldehyde-3-phosphate dehydrogenase (Yaku et al., 2018). Other possibly targeted glycolytic regulators by MAN treatment are still to be identified. Theoretically, inhibition of any glycolytic enzymes will impede glycolysis (Zhao et al., 2016; Bustamante et al., 2018; Xu et al., 2019). But in fact, these measures could be less effective in controlling inflammation. On one hand, it will lead to down-regulated TCA, and consequently reduce glucose utilization. The insufficient energy supply and glucose accumulation could bring some unfavorable consequences. On the other hand, disproportionate production of lactate cannot be addressed. From this perspective, LDH is an ideal target for the therapies of inflammatory arthritis (Li et al., 2020). Rebalancing lactate/pyruvate could be more meaningful in controlling aerobic glycolysis-fuelled inflammation (Yaromina et al., 2012). It should be noted that MAN did not simply act as a glycolic inhibitor, and it also promoted TCA. Thanks to this merit, MAN suppressed the abnormal aerobic glycolysis, and ensured the stable energy supply. In fact, the up-regulation of MAN on TCA could be similarly important to its effects on glycolysis, considering the fact that it was generally suppressed in AIA rats (Figure 5). At least, up-regulation of one presumed target KGDH is favorable for inflammation remission (Ji et al., 2020). By reshaping the metabolic profile, MAN eventually eased inflammation, and reduced energy requirement, which led to significantly improved local hypoxia.

In summary, the current study demonstrates that MAN treatment inhibited abnormal aerobic glycolysis in AIA rats partially by acting as a LDH inhibitor. Due to the slowed down ATP production, systematic inflammation was curbed, which consequently reduced the reliance on energy supply and glucose oxidation. As a result, local hypoxia and ROS accumulation were significantly improved. These changes collectively led to the down-regulation of HIF-1α/VEGF signaling pathway, and attenuated pathological angiogenesis. These findings deepen our understanding about the clinical implication of metabolism regulatory properties of MAN on RA treatment. Our previous study shows that MAN reshaped the metabolic phenotype of adipocytes, and eased systematic inflammation through their interaction with immune cells (Hu et al., 2021). This study further demonstrates that the effects of MAN on glucose metabolism are similar important in the anti-rheumatic therapies. Although the negative effects of MAN derivatives on HIF-1α/VEGF signaling have been revealed, it is the first time for us to solidly validate the anti-angiogenic potentials of MAN itself in inflammatory diseases (Kim et al., 2020). More meaningful, this work confirms that this outcome was resulted from the down-regulated aerobic glycolysis, which further highlights the metabolism regulatory properties of MAN.

## Data Availability

The original contributions presented in the study are included in the article/Supplementary Material, further inquiries can be directed to the corresponding authors.
